# Synthesis and Characterization of a Photocatalytic Material Based on Raspberry-like SiO_2_@TiO_2_ Nanoparticles Supported on Graphene Oxide

**DOI:** 10.3390/molecules28217331

**Published:** 2023-10-29

**Authors:** Citlalli Rios, L. Bazán-Díaz, Christian A. Celaya, Roberto Salcedo, Pandiyan Thangarasu

**Affiliations:** 1 Facultad de Química, Circuito Escolar s/n, Ciudad Universitaria, Universidad Nacional Autónoma de México, Coyoacán, Ciudad de México 04510, Mexico; pandiyan@unam.mx; 2 Instituto de Investigaciones en Materiales, Circuito Exterior s/n, Ciudad Universitaria, Universidad Nacional Autónoma de México, Coyoacán, Ciudad de México 04510, Mexico; bazanlulu@materiales.unam.mx (L.B.-D.); salcevitch@gmail.com (R.S.); 3 Centro de Nanociencias y Nanotecnología, Universidad Nacional Autónoma de México, Km 107 CarreteraTijuana-Ensenada, Ensenada 22800, Mexico; acelaya@iim.unam.mx

**Keywords:** TiO_2_ nanoparticles, SEM, TEM, HR-TEM, DFT analysis

## Abstract

A raspberry-like SiO_2_@TiO_2_ new material supported on functionalized graphene oxide was prepared to reduce titania’s band gap value. The material was characterized through different analytical methods such as Fourier transform infrared spectroscopy (FTIR), X-ray diffraction (XRD), scanning electron microscopy (SEM), transmission electron microscopy (TEM), and high-resolution transmission electron microscopy (HR-TEM). The band gap value was studied via UV-Vis absorption spectra and determined through the Kubelka–Munk equation. A theoretical study was also carried out to analyze the interaction between the species.

## 1. Introduction

TiO_2_ is a widely studied semiconductor material that exhibits outstanding properties such as high activity, high thermal and chemical stability, nontoxicity, low cost, and high reactivity [1,2,3]. Due to its properties, TiO_2_ has been investigated in several interesting works, one of which is the work of Honda and Fujishima [4], who studied the photocatalytic decomposition of water in 1972. After this, significant research in this field has focused on TiO_2_ nanomaterials because outstanding properties are found at this scale, specifically those related to optical and electronic behavior. Titanium is an early transition metal discovered by Gregor and Klaproth in the XVIII century [5]. This metal can be easily oxidized to yield TiO_2_. This compound is widely used due to its multiple applications. For instance, it is well-known that white paint has its pristine color from TiO_2_ dyes. Furthermore, derivatives of this substance are applied in rechargeable batteries and other electronic components.

Due to its characteristics, TiO_2_ nanomaterials have been successfully used in multiple environmental applications [6,7,8]; for example, hydrogen production through the decomposition of water, CO_2_ reduction, nitrogen fixation, and one of the most popular is photocatalysis as an advanced water treatment [9,10,11]. In this sense, TiO_2_ photocatalysis allows the elimination of substances that are hard to remove through conventional treatments [12,13], such as organic pollutants. Among those compounds are found, for instance, pharmaceuticals, personal care products (PPCPs), endocrine disruptors, etc [14,15]. The above belong to the so-called emerging pollutants [14,15,16,17], which exhibit the potential to cause damage in aqueous environments and even to human health [13,14,18].

TiO_2_ photocatalysis starts when this semiconductor is subjected to energy with a wavelength proportional to its band gap value [9,10,11]. The electrons of the valence band are excited and migrate to the conduction band $({\bar{e}}_{CB}),$ resulting in the reduction of dissolved oxygen to generate peroxide radicals. At the same time, the movement of electrons induces the formation of positive holes in the valence band $\left({h^{+}}_{VB}\right)$ where they oxidize adsorbed water to yield OH● radicals. The produced radicals participate in reactions that oxidize the organic structure of pollutants [2,3,9].

Crystalline TiO_2_ can be found in three different phases, anatase, rutile, and brookite, whose band gap energy values are 3.2 eV, 3.0 eV, and 3.4 eV, respectively [19,20,21]. Among them, anatase is the most active phase, as it exhibits higher surface adsorption to hydroxyl radicals and a longer lifetime of the electron–hole pair species (${\bar{e}}_{CB}$ and ${h^{+}}_{VB}$) [21].

Despite its advantageous properties, TiO_2_ shows some drawbacks when it comes to environmental applications. For example, it exhibits low adsorption capacity of organic pollutants, specifically those with hydrophobic characteristics [22]. Another limitation is the rapid recombination of the electron–hole pair ${(\bar{e}}_{CB}/{h^{+}}_{VB})$ that hinders the process [22,23,24] as the excited electrons return to the valence band. Additionally, the band gap energy value of TiO_2_ restricts the semiconductor to be activated only by light in the UV range of the electromagnetic spectra, corresponding to 400 nm < λ. This makes the use of specific and expensive lamps necessary to excite electrons [22,23,24]. To overcome TiO_2_ limitations, some strategies have been proposed [24,25,26]. In this sense, it has been reported that doping TiO_2_ with non-metal elements such as B, N, C, and F [25,27,28] can improve TiO_2_ performance. For instance, some studies [29,30] have reported that nitrogen-doped TiO_2_ exhibits a decrease in band gap value due to the substitution of O atoms by N atoms; this interaction modifies the electronic structure of TiO_2_ and, in addition to lowering the band gap value, it also allows TiO_2_ to absorb light at larger wavelength values [25,29,30].

Carbonaceous nanostructures have been proposed as good candidates to show exceptional features that can be used to enhance TiO_2_ properties [31]; it has been suggested that the formation of the Ti-O-C bonds modifies the band structure of TiO_2_ decreasing the band gap value and thus extending the light absorption of TiO_2_ to longer wavelengths corresponding to the visible light region. Besides the abovementioned, carbonaceous materials have been reported to improve TiO_2_ conductivity and surface area, resulting in better photocatalytic properties [25,27,28,31]. Recently, several carbon nanostructures have been used for improving TiO_2_ properties [32], for instance, carbon nanotubes, carbon quantum dots, and fullerene. Graphene and graphene oxide [31,32] have been shown to be appropriate candidates for TiO_2_ doping due to their properties, such as large surface area, high electrical and thermal conductivity, and flexible structure. Indeed, several works have stated that graphene can reduce the recombination of the photogenerated electron–hole pairs, an explanation for this is the capability of graphene to accept electrons and promote their mobility; thus, the excited electrons of TiO_2_ can easily migrate to the surface of graphene that acts as an electron sink [33,34,35,36,37], and as a consequence, reduce the recombination rate of the electron–hole pair.

Another studied strategy for improving TiO_2_ properties is the use of noble and non-noble metal ions, such as Ag, Au, Pd, Pt Cu, Fe, Co, and Ni [22,24,26]. It has been suggested that metallic species provide stability to TiO_2_ and can also form heterojunctions that modify its band structure, resulting in a lower band gap [26]. Metal oxides such as Al_2_O_3_, ZnO, ZrO_2_, MoS_2_, Fe_2_O_3_ [38], and SiO_2_ [39,40,41,42,43,44] have also been used to improve the properties of TiO_2_. In this sense, SiO_2_ is one of the most used materials because it exhibits a large surface area, low toxicity, high thermal stability, and good mechanical strength. It has been established that when joined to SiO_2_, TiO_2_ [45,46,47] exhibits enhanced thermal, chemical, and mechanical stability due to the formation of Si-O-Ti bonds [40]. Additionally, it has been demonstrated that agglomeration is avoided when using support for TiO_2_ nanoparticles such as SiO_2_ [22]. Additionally, a higher surface area is achieved, leading to better adsorption of organic molecules [41,42,43,44,48], resulting in improved photocatalytic activity.

In the present work, it is reported that the synthesis of TiO_2_ anatase nanoparticles deposited on SiO_2_ in a raspberry-like nanostructure; then, the SiO_2_@TiO_2_ system was supported on a functionalized graphene oxide matrix via covalent bonding. Therefore, the system SiO_2_@TiO_2_ is expected to be able to receive the inductive effect from the graphene oxide surface. Consequently, the final material SiO_2_@TiO_2_/GO will exhibit a significant reduction in the band gap value. The properties of the new system were studied through different techniques. A theoretical study was also carried out to analyze the interaction between SiO_2_@TiO_2_ and graphene oxide.

## 2. Results and Discussion

The main purpose of this section is to discuss the properties of the synthesized SiO_2_@TiO_2_ and SiO_2_@TiO_2_/GO materials based on different characterization techniques. The experimental results related to the modification of the band gap value of titania will be deeply discussed and compared to those theoretically obtained. This approach to studying the properties of catalysts and other battery materials has been widely used in other works [49,50,51,52].

### 2.1. XRD Characterization

**Figure 1:**
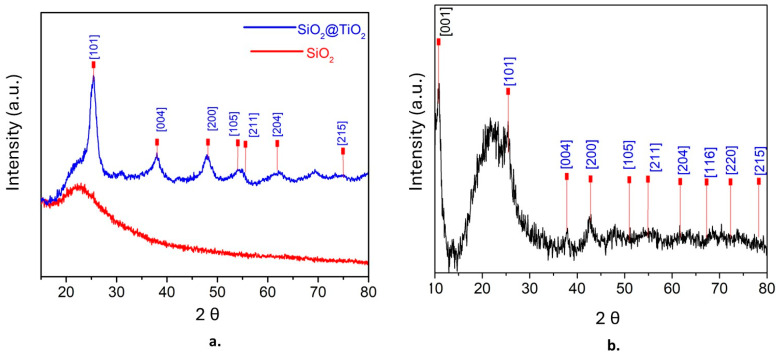
XRD patterns of (**a**) SiO_2_@TiO_2_ and (**b**) SiO_2_@TiO_2_/GO.

The powder patterns of XRD were obtained and analyzed at 2θ, first for SiO_2_@TiO_2_ and then for SiO_2_@TiO_2_/GO. Figure 1a shows the presence of amorphous silica at 2θ = 23, whereas the presence of anatase was confirmed at 25.3 [1 0 1], 37.9 [0 0 4], 47.9 [2 0 0], 54 [1 0 5], 55.5 [2 1 1], 63 [2 0 4], and 75 [2 1 5] [53]. Figure 1b shows the XRD pattern of SiO_2_@TiO_2_/GO. The peak at 2θ = 11.97 [0 0 1] corresponds to graphene oxide, and the characteristic peaks of anatase (in blue) are also evident [54]. The average grain size of TiO_2_ nanoparticles was calculated using the Scherrer equation, resulting in 8 nm.

### 2.2. FTIR Characterization

**Figure 2:**
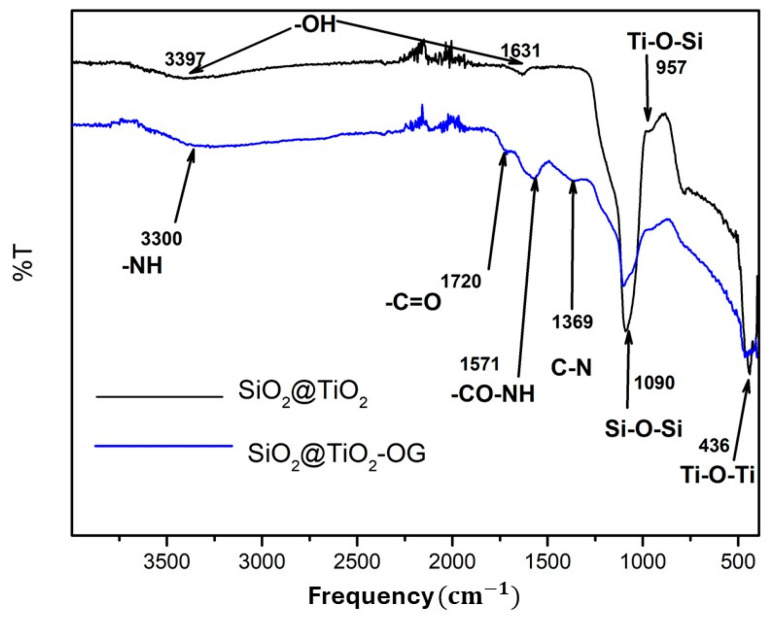
FTIR spectra of SiO_2_@TiO_2_ and SiO_2_@TiO_2_/GO.

Figure 2 shows the pattern for SiO_2_@TiO_2_ and SiO_2_@TiO_2_/GO. For SiO_2_@TiO_2,_ the bands 3397 and 1631 correspond to the stretching vibration of -OH, whereas 1090, 957, and 436 can be associated with Si-O-Si, Ti-O-Si, and Ti-O-Ti, respectively. For the SiO_2_@TiO_2_ nanoparticle supported on graphene oxide, the bands 3390, 1571, and 1369 correspond to -NH, -CO-NH, and C-N. These results indicate that the SiO_2_@TiO_2_ NPs joined to graphene oxide via covalent bonds.

### 2.3. Thermogravimetric Analysis

**Figure 3:**
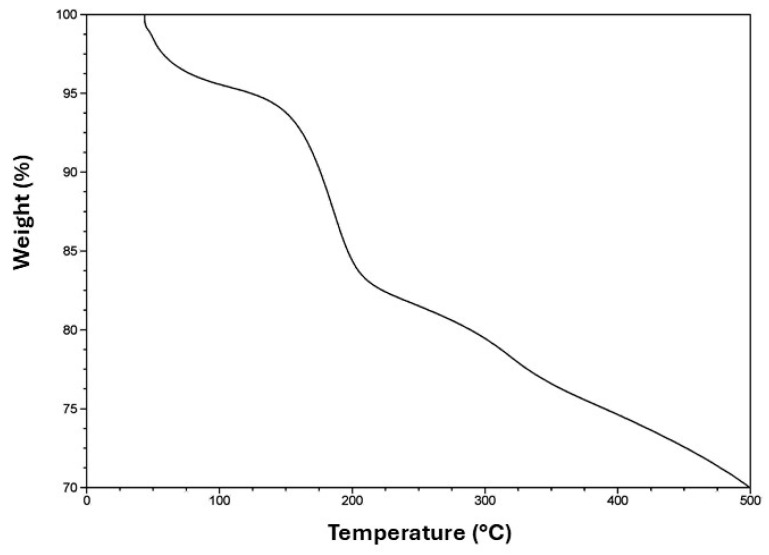
TGA curve of SiO_2_@TiO_2_/GO.

Figure 3 shows the thermogravimetric analysis for the system SiO_2_@TiO_2_/GO. The first loss of weight at 120 °C can be attributed to the water that was either chemically or physically adsorbed during the synthesis process and corresponds to approximately 5% of the material. The second loss at 200 °C can be related to the decomposition of the functional groups epoxy, hydroxy, and carboxy, which conform to the surface of graphene oxide and contribute to the bond between the surface and SiO_2_@TiO_2_. Beyond 200 °C, the weight loss is attributed only to the decomposition via pyrolysis of the carbon surface [55] to the remotion of stable oxygen groups such as phenol, carbonyl, and quinine [56].

### 2.4. SEM and HR-TEM Characterization

**Figure 4:**
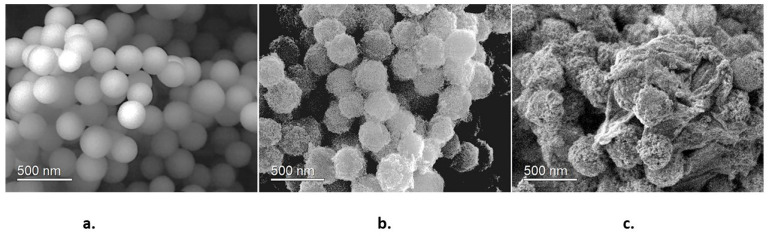
SEM images of (**a**) SiO_2_ nanoparticles, (**b**) SiO_2_@TiO_2_ raspberry-like nanoparticles, and (**c**) SiO_2_@TiO_2_/GO.

Figure 4 corresponds to SEM images of the synthesized samples. Figure 4a shows that SiO_2_ nanoparticles are homogeneous spherical particles with an average size of 250 nm. These characteristics indicate that SiO_2_ nanoparticles possess good nucleation centers for TiO_2_ nanoparticles. Figure 4b shows the SiO_2_@TiO_2_ system; it is observed that TiO_2_ nanoparticles are homogeneously dispersed on SiO_2_, yielding a raspberry-like nanoparticle. The final SiO_2_@TiO_2_/GO material is shown in Figure 4c. The joint of the nanoparticles to graphene oxide sheets is evident. This is an important result because the direct interaction between graphene oxide and TiO_2_ plays an important role in decreasing the band gap value of TiO_2_.

### 2.5. TEM and HR-TEM Analysis

**Figure 5:**
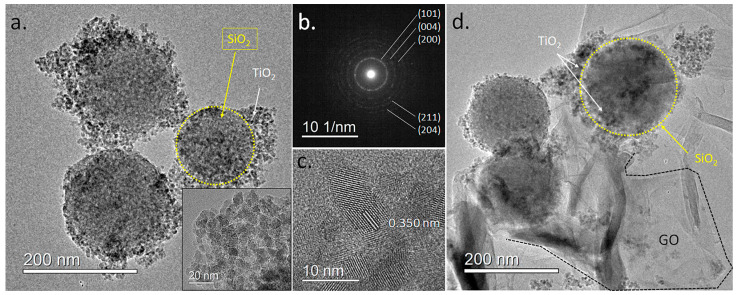
TEM images of the produced heterostructures. (**a**) Low-magnification TEM image of the SiO_2_@TiO_2_ raspberry-like nanoparticles. The inset shows a magnification of the area with TiO_2_ nanoparticles. (**b**) Electron diffraction pattern of the SiO_2_@TiO_2_ particle. (**c**) HR-TEM image of the TiO_2_ particles showing lattice fringes. (**d**) Low-magnification TEM image of the SiO_2_@TiO_2_/GO samples.

A more detailed visualization of the SiO_2_@TiO_2_ and SiO_2_@TiO_2_/GO heterostructures was obtained through TEM imaging. Figure 5a corresponds to a low-magnification TEM micrograph of isolated SiO_2_@TiO_2_ nanostructures, where the TiO_2_ nanoparticles joined to the SiO_2_ particles in a raspberry-like morphology are clearly observed (inset). The average size of the particles was 7.7 ± 1.3 nm, in accordance with the calculated crystallite size through XRD. A selected area electron diffraction (Figure 5b) performed on these particles showed a ring pattern consistent with polycrystalline TiO_2_. The measured ring diameters of 0.350, 0.237, 0.188, 0.168, and 0.148 nm can be related to the crystal planes (101), (004), (200), (211), and (204) of the anatase phase, in agreement with the XRD results.

HR-TEM imaging showed that TiO_2_ in the TiO_2_@SiO_2_ structures consisted of small monocrystalline particles, see Figure 5c. The measured lattice spacing of 0.350 nm corresponds to the (101) lattice plane of the anatase TiO_2_. The morphological features of the TiO_2_@SiO_2_/GO heterostructures were also imaged in Figure 5d. The TiO_2_@SiO_2_ particles are observed, with large GO sheets involving them to form the heterostructure.

### 2.6. UV-Vis Absorption Spectra

In a semiconductor material, the band gap value indicates the energy needed to excite an electron from the valence band to the conduction band. Determining this parameter provides information on the electronic behavior of the material and its applications. The Tauc method relates the absorption coefficient α and the band gap energy (Eg) as follows [57]:

(1) $$\alpha hv\propto {\left(hv-Eg\right)}^{n}$$

where hv is the energy of the incident photon, n can take the values ½, 3/2, 2, and 3, depending on the kind of electronic transition, Eg is the band gap, and its value is directly obtained from the plot, as the linear part of the plot is extrapolated to the *x*-axis. The Tauc method works well with samples with no light scattering; however, when working with powder samples, light scattering should be considered, and the Tauc plot cannot be applied directly [58]. In this case, diffuse reflectance spectroscopy (DRS) is a better option. In this method, the experimentally obtained reflectance is transformed through the Kubelka–Munk function into values of absorption coefficient α. As a first step, the Kubelka–Munk or reemission function $f\left(R_{\infty }\right)$ is calculated, Equation (2) [59].

(2) $$f\left(R_{\infty }\right)=\frac{K}{S}=\frac{{\left(1-R_{\infty }\right)}^{2}}{2R_{\infty }}$$

where $\left(R_{\infty }\right)=\frac{R_{Sample}}{R_{Reference}}$ is the reflectance from an infinitely thick specimen, K represents the absorption coefficient, and S is the scattering coefficient.

Finally, the calculated $f\left(R_{\infty }\right)$ can take the place of α in the Tauc equation as follows:

(3) $$f\left(R_{\infty }\right)hv\propto {\left(hv-Eg\right)}^{n}$$

This work measured diffusion reflectance for the samples in a UV-Vis Cary 5000 spectrophotometer at a wavelength ranging from 700 to 200 nm. Results were analyzed via the Kubelka–Munk method. The band gap was then obtained through the Tauc plot.

**Figure 6:**
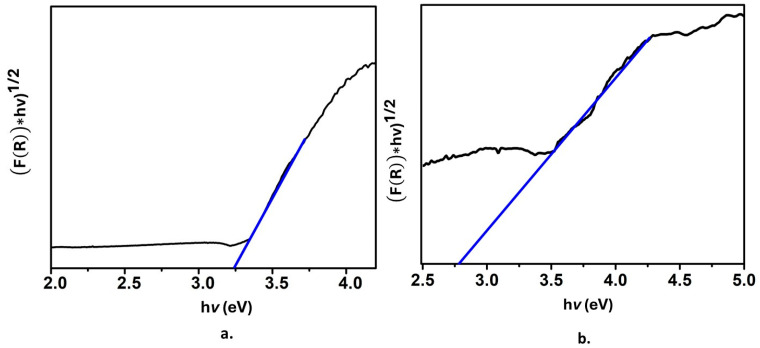
Tauc plot of (**a**) SiO_2_@ TiO_2_ raspberry-like nanoparticles and (**b**) SiO_2_@TiO_2_ supported on GO.

Figure 6a shows the Tauc plot for the SiO_2_@TiO_2_ system; the band gap value 3.2 eV corresponds to the TiO_2_ anatase phase, while Figure 6b shows the Tauc plot for SiO_2_@TiO_2_/GO. In this case, the band gap value is 2.7 eV. This is a remarkable result; as previously mentioned, TiO_2_ exhibits some drawbacks related to the position of its conduction and valence bands that define the band gap value and, at the same time, restrict TiO_2_ from being activated with the energy of only a portion of the electromagnetic spectra, the UV region and, as a consequence, limits its application. The reported decrease in the band gap value indicates that the presence of graphene oxide modifies the band structure of TiO_2_ since graphene oxide exhibits high conductivity, and its band gap is narrower than that of anatase. Thus, when associating graphene oxide and the raspberry-like nanoparticles, the highly populated HOMO from graphene oxide favors the attraction of virtual molecular orbitals of it and those coming from TiO_2_. It is expected that the decrease of the band gap provokes a value that shifts the adsorption range of TiO_2_ to a higher wavelength corresponding to the visible portion of the spectrum, which also enlarges the possible applications of the material.

Another expected improvement is related to the high electron mobility exhibited by graphene oxide, as this species is well-known due to the π–π interactions taking place in its structure. The functional groups such as amine, epoxy, and hydroxyl provoke an inductive effect that improves the electron mobility of graphene oxide [60]. Thus, the excited electrons of TiO_2_ are easily transferred to graphene oxide, avoiding the fast recombination of those species and conferring titania a better photocatalytic activity [61]. The nature of those interactions will be discussed in the theoretical section.

### 2.7. Theoretical Studies

**Figure 7:**
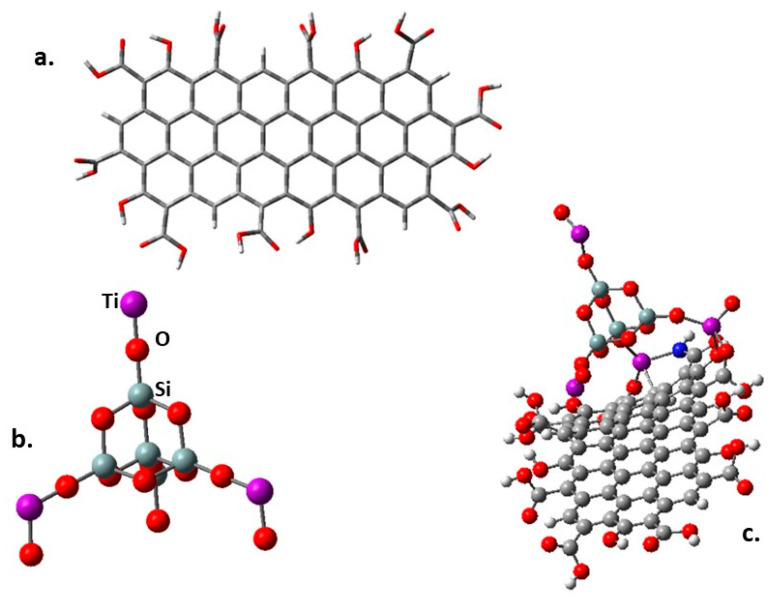
(**a**) The fragment of functionalized graphene oxide. (**b**) SiO_2_@TiO_2_ raspberry-like nanoparticle used in the present simulation. (**c**) SiO_2_@TiO_2_ raspberry-like nanoparticle supported on graphene oxide.

The band gap behavior was also studied through a theoretical DFT method. The molecular structure of all the involved species was optimized using the CAM-B3LYP method [62] included in the Gaussian 16 pack [63], and the calculations were carried out using the basis set 6-31g**. The stable minimum energy configuration of each molecule was approached by calculating their frequencies. The design of the fragment of modified GO is shown in Figure 7a.

There are two optimized structures to consider. The first one is shown in Figure 7a, where all the peripheral substituents are carboxylic groups and a species where some amine -NH_2_ group replaces the -OH of some carboxylic groups. This last possibility was studied because there are reports using this functionalization to link this kind of nanoparticles to graphene derivatives [64], and even more importantly, this is the synthesis followed in this work. Furthermore, a variation of the graphene itself consists of placing epoxy groups on the inner surface, searching for interaction with terminal titanium atoms. The designed structure shown in Figure 7b was conceived following other interesting propositions [65] where the stability and chemical properties of mixed Ti and Si oxides are tested. Some Si atoms of a SiO_2_ crystal cell were substituted by Ti atoms. Figure 7c shows the first simulation, which involves the SiO_2_@TiO_2_ raspberry-like nanoparticle interacting on the periphery of the functionalized graphene oxide derivative.

A fact to highlight is that the interaction among titanium atoms from the nanoparticle and the carboxylated graphene oxide peripheral groups can occur either on oxygen or nitrogen terminals. They are real coordinated covalent bonds with the lengths 2.04 Å and 2.08 Å, respectively. The found values match well with the reported lengths for this kind of bond [66,67]; additionally, they show Wiberg indexes of 0.552 and 0.511, respectively, which suggests a real bond [68].

The purpose of studying the interaction between TiO_2_ and other compounds is to significantly reduce titania´s band gap value. With that in mind, the calculated band gap of the alone mixed-oxide species and the same for the GO-TiSi complex were calculated in the same conditions. The first one yields a value of 3.9 eV, but the same value for the complex is 2.3 eV. This is a demonstration of the validity of the proposition; the last value experiment decreases nearly half of the original one.

**Figure 8:**
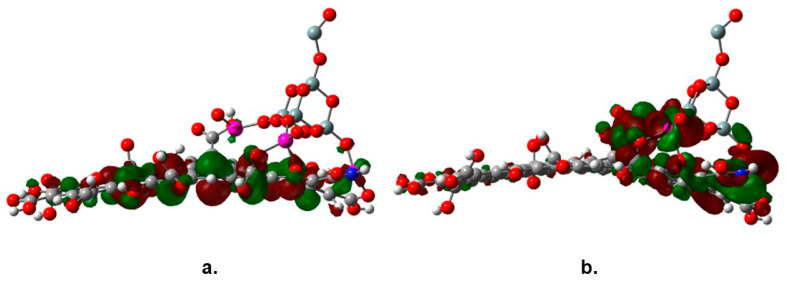
Frontier molecular orbitals (**a**) HOMO and (**b**) LUMO of SiO_2_@TiO_2_/GO.

The observed decrease of the band gap value of TiO_2_ is the result of the interaction of the molecular orbitals of both joined structures, graphene oxide and the SiO_2_@TiO_2_ raspberry-like nanoparticles. One feature that has been signaled about the nature of the association between functionalized graphene and nanoparticles of a catalyst is the new behavior of the species as a semiconductor; in fact, the new complex combines the characteristics of both parent molecules. Regarding the energy gap, a peculiar phenomenon is observed; the SiO_2_@TiO_2_ raspberry-like nanoparticle is expected to be a semiconductor itself with a discrete HOMO-LUMO set; however, these frontier molecular orbitals experiment a notorious change when the large reservoir of orbitals coming from the graphene oxide influence on them. Figure 8 shows the frontier molecular orbitals of the studied species, the HOMO in Figure 8a shows a strong influence of graphene oxide, whereas the LUMO in Figure 8b is focused on titanium atoms; this arrangement is one of the main reasons leading to the change in the energy gap value.

## 3. Materials and Methods

### 3.1. Chemicals

There are different precursors for tetraethyl orthosilicate TEOS, titanium isopropoxide TTIP, NH_4_OH, HCl, EtOH, APTES, NaOH, and ClCH_2_COONa (Sigma–Aldrich, St. Louis, MO, USA) were used as received. Graphene oxide (Sigma–Aldrich) was modified to create more active sites.

### 3.2. Synthesis

#### 3.2.1. SiO_2_ Nanoparticles

The sol–gel synthesis of SiO_2_ nanoparticles starts with a hydrolysis reaction of alkoxy groups contained in the precursor. In this work, tetraethyl orthosilicate was chosen as a precursor because it contains enough alkoxy groups to hydrolysate [69]. According to the synthesis established by Stöber [70], the process started with the hydrolysis of TEOS in basic media and was followed by a series of condensation and hydrolysis reactions to finally obtain the desired SiO_2_ nanoparticles. Briefly, 3 mL of TEOS was quickly added to a solution of NH_4_, H_2_O, and ethanol (4 mL:15 mL:100 mL) and left under magnetic stirring for 1 h. The mixture was neutralized with HCl. The result was centrifuged at 3500 rpm for 10 min. The precipitate was washed with water and dried at 70 °C for 20 h.

#### 3.2.2. SiO_2_@TiO_2_ Raspberry-like Nanoparticles

TiO_2_ nanoparticles were also obtained via a sol–gel synthesis; again, a precursor containing alkoxy groups was chosen. In this case, the process started with the hydrolyzation reaction of titanium isopropoxide (TTiP) and was followed by condensation and hydrolysis reactions. A total of 0.2 g of SiO_2_ NPs was dispersed in 15 mL of isopropanol and left under magnetic stirring. On the other hand, TTiP was dissolved in EtOH and was slowly added (approximately 1 mL/min) to the dispersion. The mixture was left under magnetic stirring for 20 h. Then, a solution of water–isopropanol (3 mL:6 mL) was added to promote hydrolysis and condensation reactions. The result was left under magnetic stirring for 2 h. Then, it was transferred to a Teflon reactor for a thermic treatment 24 h, 10 °C. The product was recovered via centrifugation, washed with water, and dried for 24 h at 100 °C. The product was calcined at 500 °C for 3 h to obtain the anatase phase of TiO_2_ [43,44].

#### 3.2.3. SiO_2_@TiO_2_ Raspberry-like Nanoparticles Supported on Graphene Oxide

**Figure 9:**
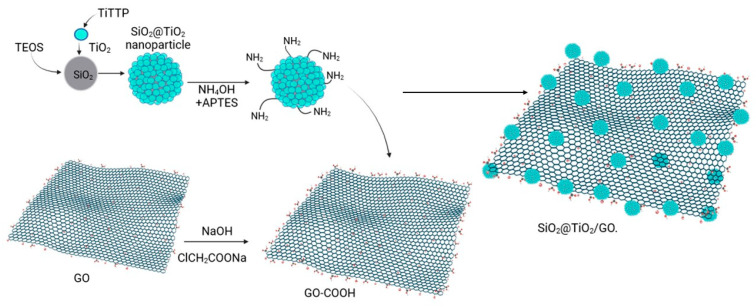
Synthesis of SiO_2_@TiO_2_ nanoparticles supported on functionalized graphene oxide (Created with BioRender.com).

SiO_2_@TiO_2_ raspberry-like nanoparticles were supported on graphene oxide through chemical bonding. It is said that once obtained, the system SiO_2_@TiO_2_ and the graphene oxide layer were functionalized to create the appropriate sites for the bonding [71]. SiO_2_@TiO_2_ NPs were suspended in a mixture of 20 mL of EtOH and 4 mL of deionized water. Then, 0.5 mL of NH_4_OH and 50 µL of APTES were added to create the amino (-NH_2_) group over the SiO_2_@TiO_2_ surface. Furthermore, it was left under magnetic stirring for 24 h. The product SiO_2_@TiO_2_ -NH_2_ was washed with EtOH and dried at 60 °C for 2 h. The graphene oxide surface was modified to obtain a more carboxylic acid group species GO-COOH. Graphene oxide was suspended in water using ultrasonication, NaOH and ClCH_2_COONa were added, and the reaction was left under magnetic stirring for 3 h. The product was washed using a dialysis tube for 72 h. The precipitate was dried at 90 °C for 12 h. To obtain the species SiO_2_@TiO_2_/GO, a mixture of 20 mL of deionized water and 20 mg of EDC-HCl was prepared, and 10 mg of GO-COOH was dispersed using ultrasonication for 2 h. A total of 10 mg of SiO_2_@ TiO_2_-NH_2_ was added, and the reaction was left under magnetic stirring for 24 h. The product was washed with deionized water and EtOH and dried at 60 °C for 12 h [48]. Figure 9 shows the steps followed to obtain the SiO_2_@TiO_2_ raspberry-like nanoparticles and the final species SiO_2_@TiO_2_/GO.

### 3.3. Characterization

The crystalline structure of the nanoparticles was studied using X-ray diffraction (XRD), and the spectra were obtained to analyze the crystalline phases of the samples using a Bruker D8 Advance diffractometer with Cu-Kα radiation (λ = 0.154 nm). The average size of the crystal lattices of TiO_2_ was calculated using Scherrer’s formula [72]. Fourier transform infrared spectra were collected on a Nicolet 510P spectrometer.

To characterize the crystalline structure, morphology, and average diameter of the obtained compounds, a scanning electron microscope (SEM) and a high-resolution transmission electron microscope (HR-TEM) analysis were performed using a JEOL 7600F and a JEOL ARM200F operated at 20 keV, respectively. The electronic diffusion spectra were obtained using a UV-Vis Cary 5000 spectrophotometer, and the band gap was determined through Tauc’s plot using the Kubelka–Munk method. Thermogravimetric analysis, TGA, was performed using a TGA Q5000 V3.17 Build 265 equipment in a nitrogen atmosphere.

## 4. Conclusions

A new nanomaterial based on SiO_2_@TiO_2_ nanoparticles supported on graphene oxide was synthesized and characterized through XRD, FTIR, SEM, and HR-TEM. The band gap value was also determined using the Kubelka–Munk method.

The SiO_2_@TiO_2_ nanoparticle exhibits a raspberry-like morphology, suggesting that TiO_2_ nanoparticles are exposed in such a way that they can be considered for catalytic processes. It was found that when joining SiO_2_@TiO_2_ to graphene oxide layers, the band gap of TiO_2_ was lowered by almost 1 eV with respect to the band gap value of anatase. This result indicates that the conductor properties of graphene oxide endow TiO_2_ with new properties.

Theoretical calculations of discrete molecules were carried out to evaluate the improvement of the electronic activity using graphene surfaces. The theoretical band gap value is in agreement with the experimental one, validating the research base.

## Data Availability

Not applicable.

## Sample Availability

Not available.

## References

[1] Nakata K., Fujishima A. (2012). TiO_2_ photocatalysis: Design and applications. J. Photochem. Photobiol. C Photochem. Rev..

[2] Henderson M.A. (2011). A surface science perspective on TiO_2_ photocatalysis. Surf. Sci. Rep..

[3] Fujishima A., Zhang X., Tryk D.A. (2008). TiO_2_ photocatalysis and related surface phenomena. Surf. Sci. Rep..

[4] Fujishima A., Honda K. (1972). Electrochemical Photolysis of Water at a Semiconductor Electrode. Nature.

[5] Enghag P. (2004). Titanium. Encyclopedia of the Elements.

[6] Kang X., Liu S., Dai Z., He Y., Song X., Tan Z. (2019). Titanium Dioxide: From Engineering to Applications. Catalysts.

[7] Truppi A., Petronella F., Placido T., Striccoli M., Agostiano A., Curri M.L., Comparelli R. (2017). Visible-Light-Active TiO_2_-Based Hybrid Nanocatalysts for Environmental Applications. Catalysts.

[8] Li Z., Wang S., Wu J., Zhou W. (2022). Recent progress in defective TiO_2_ photocatalysts for energy and environmental applications. Renew. Sustain. Energy Rev..

[9] Herrmann J.M., Guillard C., Pichat P. (1993). Heterogeneous photocatalysis: An emerging technology for water treatment. Catal. Today.

[10] Herrmann J.M. (1999). Heterogeneous photocatalysis: Fundamentals and applications to the removal of various types of aqueous pollutants. Catal. Today.

[11] Pelaez M., Nolan N.T., Pillai S.C., Seery M.K., Falaras P., Kontos A.G., Dunlop P.S., Hamilton J.W., Byrne J.A., O’shea K. (2012). A review on the visible light active titanium dioxide photocatalysts for environmental applications. Appl. Catal. B.

[12] Geissen V., Mol H., Klumpp E., Umlauf G., Nadal M., van der Ploeg M., van de Zee S.E., Ritsema C.J. (2015). Emerging pollutants in the environment: A challenge for water resource management. Int. Soil Water Conserv. Res..

[13] Arman N.Z., Salmiati S., Aris A., Salim M.R., Nazifa T.H., Muhamad M.S., Marpongahtun M. (2021). A Review on Emerging Pollutants in the Water Environment: Existences, Health Effects and Treatment Processes. Water.

[14] Gogoi A., Mazumder P., Tyagi V.K., Tushara Chaminda G.G., An A.K., Kumar M. (2018). Occurrence and fate of emerging contaminants in water environment: A review. Groundw. Sustain. Dev..

[15] Wilkinson J., Hooda P.S., Barker J., Barton S., Swinden J. (2017). Occurrence, fate and transformation of emerging contaminants in water: An overarching review of the field. Environ. Pollut..

[16] Kabir E.R., Rahman M.S., Rahman I. (2015). A review on endocrine disruptors and their possible impacts on human health. Environ. Toxicol. Pharmacol..

[17] Méndez E., González-Fuentes M.A., Rebollar-Perez G., Méndez-Albores A., Torres E. (2017). Emerging pollutant treatments in wastewater: Cases of antibiotics and hormones. J. Environ. Sci. Health Part A.

[18] Vasilachi I.C., Asiminicesei D.M., Fertu D.I., Gavrilescu M. (2021). Occurrence and Fate of Emerging Pollutants in Water Environment and Options for Their Removal. Water.

[19] Reyes-Coronado D., Rodríguez-Gattorno G., Espinosa-Pesqueira M.E., Cab C., De Coss R., Oskam G. (2008). Phase-pure TiO_2_ nanoparticles: Anatase, brookite and rutile. Nanotechnology.

[20] Landmann M., Rauls E., Schmidt W.G. (2012). The electronic structure and optical response of rutile, anatase and brookite TiO_2_. J. Phys. Condens. Matter.

[21] Zhang J., Zhou P., Liu J., Yu J. (2014). New understanding of the difference of photocatalytic activity among anatase, rutile and brookite TiO_2_. Phys. Chem. Chem. Phys..

[22] Dong H., Zeng G., Tang L., Fan C., Zhang C., He X., He Y. (2015). An overview on limitations of TiO_2_-based particles for photocatalytic degradation of organic pollutants and the corresponding countermeasures. Water Res..

[23] Etacheri V., Di Valentin C., Schneider J., Bahnemann D., Pillai S.C. (2015). Visible-light activation of TiO_2_ photocatalysts: Advances in theory and experiments. J. Photochem. Photobiol. C Photochem. Rev..

[24] Park H., Park Y., Kim W., Choi W. (2013). Surface modification of TiO_2_ photocatalyst for environmental applications. J. Photochem. Photobiol. C Photochem. Rev..

[25] Basavarajappa P.S., Patil S.B., Ganganagappa N., Reddy K.R., Raghu A.V., Reddy C.V. (2020). Recent progress in metal-doped TiO_2_, non-metal doped/codoped TiO_2_ and TiO_2_ nanostructured hybrids for enhanced photocatalysis. Int. J. Hydrogen Energy.

[26] Fang W., Xing M., Zhang J. (2017). Modifications on reduced titanium dioxide photocatalysts: A review. J. Photochem. Photobiol. C Photochem. Rev..

[27] Hodaifa G., Albqmi M., Akhter P., Arshad A., Saleem A., Hussain M. (2022). Recent Development in Non-Metal-Doped Titanium Dioxide Photocatalysts for Different Dyes Degradation and the Study of Their Strategic Factors: A Review. Catalysts.

[28] Piątkowska A., Janus M., Szymański K., Mozia S. (2021). C-,n-and s-doped tio_2_ photocatalysts: A review. Catalysts.

[29] Ansari S.A., Khan M.M., Ansari M.O., Cho M.H. (2016). Nitrogen-doped titanium dioxide (N-doped TiO_2_) for visible light photocatalysis. New J. Chem..

[30] Bakar S.A., Ribeiro C. (2016). Nitrogen-doped titanium dioxide: An overview of material design and dimensionality effect over modern applications. J. Photochem. Photobiol. C Photochem. Rev..

[31] Leary R., Westwood A. (2011). Carbonaceous nanomaterials for the enhancement of TiO_2_ photocatalysis. Carbon N. Y..

[32] Ghumro S.S., Lal B., Pirzada T. (2022). Visible-Light-Driven Carbon-Doped TiO_2_-Based Nanocatalysts for Enhanced Activity toward Microbes and Removal of Dye. ACS Omega.

[33] Ferrighi L., Fazio G., Di Valentin C., Ferrighi L., Fazio G., Di Valentin C. (2016). Charge Carriers Separation at the Graphene/(101) Anatase TiO_2_ Interface. Adv. Mater. Interfaces.

[34] Du A., Ng Y.H., Bell N.J., Zhu Z., Amal R., Smith S.C. (2011). Hybrid graphene/titania nanocomposite: Interface charge transfer, hole doping, and sensitization for visible light response. J. Phys. Chem. Lett..

[35] Li J., Zhou S.L., Hong G.B., Chang C.T. (2013). Hydrothermal preparation of P25–graphene composite with enhanced adsorption and photocatalytic degradation of dyes. Chem. Eng. J..

[36] Trapalis A., Todorova N., Giannakopoulou T., Boukos N., Speliotis T., Dimotikali D., Yu J. (2016). TiO_2_/graphene composite photocatalysts for NOx removal: A comparison of surfactant-stabilized graphene and reduced graphene oxide. Appl. Catal. B.

[37] Jo W.K., Kang H.J. (2013). Titanium dioxide–graphene oxide composites with different ratios supported by Pyrex tube for photocatalysis of toxic aromatic vapors. Powder Technol..

[38] Fawzi Suleiman Khasawneh O., Palaniandy P. (2021). Removal of organic pollutants from water by Fe_2_O_3_/TiO_2_ based photocatalytic degradation: A review. Environ. Technol. Innov..

[39] Shul Y.G., Kim H.J., Haam S.J., Han H.S. (2003). Photocatalytic characteristics of TiO_2_ supported on SiO_2_. Res. Chem. Intermed..

[40] Permpoon S., Houmard M., Riassetto D., Rapenne L., Berthomé G., Baroux B., Joud J.C., Langlet M. (2008). Natural and persistent superhydrophilicity of SiO_2_/TiO_2_ and TiO_2_/SiO_2_ bi-layer films. Thin Solid Films.

[41] Wilhelm P., Stephan D. (2007). Photodegradation of rhodamine B in aqueous solution via SiO_2_@TiO_2_ nano-spheres. J. Photochem. Photobiol. A Chem..

[42] Wu J., Wang H., Bao L., Zhong J., Chen R., Sun L. (2018). Novel raspberry-like hollow SiO_2_@TiO_2_ nanocomposites with improved photocatalytic self-cleaning properties: Towards antireflective coatings. Thin Solid Films.

[43] Li X., He J. (2013). Synthesis of raspberry-like SiO_2_-TiO_2_ nanoparticles toward antireflective and self-cleaning coatings. ACS Appl. Mater. Interfaces.

[44] Ullah S., Ferreira-Neto E.P., Pasa A.A., Alcântara C.C., Acuña J.J., Bilmes S.A., Ricci M.L.M., Landers R., Fermino T.Z., Rodrigues-Filho U.P. (2015). Enhanced photocatalytic properties of core@shell SiO_2_@TiO_2_ nanoparticles. Appl. Catal. B.

[45] Chun H., Yizhong W., Hongxiao T. (2001). Influence of adsorption on the photodegradation of various dyes using surface bond-conjugated TiO_2_/SiO_2_ photocatalyst. Appl. Catal. B.

[46] Mahanta U., Khandelwal M., Deshpande A.S. (2022). TiO_2_@SiO_2_ nanoparticles for methylene blue removal and photocatalytic degradation under natural sunlight and low-power UV light. Appl. Surf. Sci..

[47] Pakdel E., Daoud W.A., Seyedin S., Wang J., Razal J.M., Sun L., Wang X. (2018). Tunable photocatalytic selectivity of TiO_2_/SiO_2_ nanocomposites: Effect of silica and isolation approach. Colloids Surf. A Physicochem. Eng. Asp..

[48] Chen F., Yan F., Chen Q., Wang Y., Han L., Chen Z., Fang S. (2014). Fabrication of Fe_3_O_4_@SiO_2_@TiO_2_ nanoparticles supported by graphene oxide sheets for the repeated adsorption and photocatalytic degradation of rhodamine B under UV irradiation. Dalton Trans..

[49] Li W., Xu H., Zhang H., Wei F., Huang L., Ke S., Fu J., Jing C., Cheng J., Liu S. (2023). Tuning electron delocalization of hydrogen-bonded organic framework cathode for high-performance zinc-organic batteries. Nat. Commun..

[50] Yang Y., Sun M., Chen Z., Xu H., Wang X., Duan J., Hou B. (2023). 3D nanothorn cluster-like Zn-Bi2S3 sensitized WO_3_/ZnO multijunction with electron-storage characteristic and adjustable energy band for improving sustained photoinduced cathodic protection application. Chem. Eng. J..

[51] Guan D., Xu H., Zhang Q., Huang Y.C., Shi C., Chang Y.C., Xu X., Tang J., Gu Y., Pao C.W. (2023). Identifying a Universal Activity Descriptor and a Unifying Mechanism Concept on Perovskite Oxides for Green Hydrogen Production. Adv. Mater..

[52] Lv Z., Xu H., Xu W., Peng B., Zhao C., Xie M., Lv X., Gao Y., Hu K., Fang Y. (2023). Quasi-Topological Intercalation Mechanism of Bi_0.67_NbS_2_ Enabling 100 C Fast-Charging for Sodium-Ion Batteries. Adv. Energy Mater..

[53] AMCSD Search Results. http://rruff.geo.arizona.edu/AMS/minerals/Anatase.

[54] Gebreegziabher G.G., Asemahegne A.S., Ayele D.W., Dhakshnamoorthy M., Kumar A. (2019). One-step synthesis and characterization of reduced graphene oxide using chemical exfoliation method. Mater. Today Chem..

[55] Farivar F., Lay Yap P., Karunagaran R.U., Losic D. (2021). Thermogravimetric Analysis (TGA) of Graphene Materials: Effect of Particle Size of Graphene, Graphene Oxide and Graphite on Thermal Parameters. C.

[56] Chang B.Y.S., Huang N.M., An’amt M.N., Marlinda A.R., Norazriena Y., Muhamad M.R., Harrison I., Lim H.N., Chia C.H. (2012). Facile hydrothermal preparation of titanium dioxide decorated reduced graphene oxide nanocomposite. Int. J. Nanomed..

[57] Tauc J., Grigorovici R., Vancu A. (1966). Optical Properties and Electronic Structure of Amorphous Germanium. Phys. Status Solidi B.

[58] Makuła P., Pacia M., Macyk W. (2018). How To Correctly Determine the Band Gap Energy of Modified Semiconductor Photocatalysts Based on UV-Vis Spectra. J. Phys. Chem. Lett..

[59] Kubelka P. (1948). New Contributions to the Optics of Intensely Light-Scattering Materials. Part, I. JOSA.

[60] Abid S.P., Islam S.S., Mishra P., Ahmad S. (2018). Reduced graphene oxide (rGO) based wideband optical sensor and the role of Temperature, Defect States and Quantum Efficiency. Sci. Rep..

[61] Li L., Yu L., Lin Z., Yang G. (2016). Reduced TiO_2_-Graphene Oxide Heterostructure As Broad Spectrum-Driven Efficient Water-Splitting Photocatalysts. ACS Appl. Mater. Interfaces.

[62] Caldeweyher E., Bannwarth C., Grimme S. (2017). Extension of the D3 dispersion coefficient model. J. Chem. Phys..

[63] Frisch M.J., Trucks G.W., Schlegel H.B., Scuseria G.E., Robb M.A., Cheeseman J.R. (2016). Gaussian 16.

[64] Wanag A., Kapica-Kozar J., Sienkiewicz A., Rokicka-Konieczna P., Kusiak-Nejman E., Morawski A.W. (2022). Preliminary Findings on CO_2_ Capture over APTES-Modified TiO_2_. Atmosphere.

[65] Cuko A., Calatayud M., Bromley S.T. (2018). Stability of mixed-oxide titanosilicates: Dependency on size and composition from nanocluster to bulk. Nanoscale.

[66] Wright D.A., Williams D.A. (1968). The Crystal and Molecular Structure of Titanium Tetramethoxide. Acta Cryst..

[67] Yu S., Zeng Q., Oganov A.R., Frapper G., Zhang L. (2015). Phase stability, chemical bonding and mechanical properties of titanium nitrides: A first-principles study. Phys. Chem. Chem. Phys..

[68] Zouchoune B. (2018). Stability and possible multiple metal-metal bonding in tetranuclear sandwich complexes of cyclooctatetraene ligand. Struct. Chem..

[69] Heuer-Jungemann A., Feliu N., Bakaimi I., Hamaly M., Alkilany A., Chakraborty I., Masood A., Casula M.F., Kostopoulou A., Oh E. (2019). The role of ligands in the chemical synthesis and applications of inorganic nanoparticles. Chem. Rev..

[70] Stöber W., Fink A., Bohn E. (1968). Controlled growth of monodisperse silica spheres in the micron size range. J. Colloid. Interface Sci..

[71] He F., Fan J., Ma D., Zhang L., Leung C., Chan H.L. (2010). The attachment of Fe_3_O_4_ nanoparticles to graphene oxide by covalent bonding. Carbon N. Y..

[72] Scherrer P. (1918). Bestimmung der Grösse und der inneren Struktur von Kolloidteilchen mittels Röntgenstrahlen. Nachrichten Von Der Ges. Der Wiss. Zu Göttingen Math.-Phys. Kl..

